# American Indian chronic Renal insufficiency cohort study (AI-CRIC study)

**DOI:** 10.1186/s12882-020-01954-y

**Published:** 2020-07-22

**Authors:** Mark L. Unruh, Soraya Arzhan, Harold I. Feldman, Helen C. Looker, Robert G. Nelson, Thomas Faber, David Johnson, Linda Son-Stone, Vernon S. Pankratz, Larissa Myaskovsky, Vallabh O. Shah, the CRIC study investigators, Lawrence J. Appel, Alan S. Go, Jiang He, James P. Lash, Mahboob Rahman, Panduranga S. Rao, Raymond R. Townsend

**Affiliations:** 1 Department of Internal Medicine and Biochemistry, University of New Mexico, School of Medicine, MSC 10 5550, Albuquerque, NM 87131 USA; 2 University of Pennsylvania, Philadelphia, PA USA; 3 NIDDK, NIH, Phoenix, AZ USA; 4 Indian Health Services, Zuni, NM USA; 5 First Nations Hospital, Albuquerque, NM USA

**Keywords:** Chronic kidney disease, Cardiovascular disease, American Indians, AI-CRIC, End stage Renal disease, Environmental exposure, eGFR, Heart rate variability

## Abstract

**Background:**

Chronic kidney disease (CKD) is an increasing epidemic globally that is associated with adverse health outcomes including end stage kidney disease (ESKD), cardiovascular disease (CVD), and death. American Indians (AIs) have a higher prevalence of CKD than most other racial/ethnic groups, due in part to a high prevalence of type 2 diabetes. Other genetic and environmental factors not yet identified may also contribute to the disproportionate burden of CKD in AIs.

**Method:**

We will establish 3 clinical centers to recruit AIs from the Southwest United States (US) to expand the Chronic Renal Insufficiency Cohort (CRIC) study. We will follow the current CRIC protocol for kidney and cardiovascular measures and outcomes, which include ambulatory monitoring of kidney function and the use of mobile health technologies for CVD sub-phenotyping, and compare the outcomes in AIs with those in other racial/ethnic groups in CRIC.

**Discussion:**

AI-CRIC will identify the role of various risk factors for rapid loss of kidney function among AIs of the Southwest US. In addition, to better understand the natural history of CKD and CVD in this high-risk population, we will identify unique risk factors for CKD and CVD progression in AIs. We will also compare event rates and risk factors for kidney and cardiovascular events in AIs with the other populations represented in CRIC.

## Background

Chronic kidney disease (CKD) is an increasing epidemic affecting people globally [1]. The prevalence of CKD among US adults is estimated at just under 15% [2], since American Indians (AIs) in the Southwest US have a prevalence of mild to moderate CKD of 17% [3]. Associated morbidity, mortality and economic burden mostly derive from progression to end stage kidney disease (ESKD) and cardiovascular disease (CVD). AIs suffer from substantial health disparities in the context of broad historical and contemporary social and economic inequality [3]. Risk factors for diabetic kidney disease, the predominant form of CKD among AIs, include the traditional factors such as hyperglycemia, hypertension, and inheritance, but may also include other factors such as exposure to various persistent environmental pollutants. In addition, the increasing prevalence of obesity in AI youth threatens to exacerbate the burden of CKD in these communities because the earlier onset of diabetes is associated with an increase in albuminuria, CKD, and ESKD in mid-life [4, 5]. Although the degree of proteinuria reduction from use of renin-angiotensin-aldosterone system inhibitors (RAASi) correlates with subsequent reduction in the rate of ESKD [6, 7], there are currently no markers available to predict the subsequent loss of kidney function in AI patients already treated with these agents. In addition to continuing the search for circulating and genetic markers of CKD progression, new paradigms have emerged to identify the role of subclinical risk factors (e.g., Cystatin C) for rapid loss of kidney function among AIs. Measurement of kidney function variability over time and detection of acute decrements in kidney function will be an important focus of the AI-CRIC Study. Several recent clinical trials examining treatments to slowing down CKD progression have failed to show outcome benefit in part because of the inability to identify prospectively, and randomize those at greatest risk of rapid progression while receiving standard of care treatment [8, 9].

CKD among AIs needs to be studied more deeply in order to answer many questions about the progression of CKD among AIs, as well as the prevalence of CVD in the setting of CKD in these populations. Assessment of morbidity and mortality associated with CKD, often from CVD, requires long-term study of affected individuals, and highlights the public health importance of this research initiative [10]. To address this burden of CKD in AI communities, our CRIC ancillary cohort study of 500 AIs (AI-CRIC) will improve our understanding of both potential risk factors for CKD progression, and the scope of this disease among AIs. Our study was designed to accomplish the following specific aims:

1) Identify unique risk factors for CKD and CVD progression and compare CKD and CVD event rates and risk factors between AIs and the other populations represented in CRIC.

2) Using an important paradigm shift for monitoring kidney function, we will conduct ambulatory monitoring of kidney function and damage using a handheld device analogous to a glucometer, and evaluate its relationship with exposure data.

3) Using mobile health technologies, we will conduct CVD sub-phenotyping and evaluate its relationship with exposures data.

Our study leverages the strengths of the current CRIC study and incorporates the planned activities of the next phase of the study by implementing contemporary CRIC protocols for kidney and cardiovascular measurements and outcomes in AIs.

## Methods/design

### Study overview

The overall goals of the proposed prospective cohort study are to: (a) precisely assess the extent to which the rates of CKD and CVD progression differ between AIs and other racial/ethnic groups based on standardized definitions used throughout CRIC; and, (b) to identify the association of CKD to the levels of potential environmental and occupational exposures unique to AIs residing in the American Southwest. To better understand the natural history of CKD/CVD in this high-risk population, we will implement the CRIC 2018 protocol of ambulatory monitoring of kidney function and damage along with evaluations of CVD sub-phenotypes using mobile health technologies. Data collection will take place at three sites located across the Southwest US. We will adopt the general methods and procedures of the parent CRIC protocol (http://www.cristudy.org).

We obtained Letters of Support from Zuni Comprehensive Community Health Center - Indian Health Services, First Nations Community Health Source, Pueblo of Zuni Tribal Governor, and NIDDK, NIH Phoenix.

### Inclusion and exclusion criteria

**Inclusion Criteria: Age:** Eligible age at enrollment is 21 to 80 years, which will ensure a sufficient number of CVD outcome events and statistical power while minimizing the impact of competing risks (deaths due to non-renal /non-CVD causes) and dropout. **Diabetes Status:** We will use the criteria of The Expert Committee on the Diagnosis and Classification of Diabetes Mellitus to diagnose diabetes status. **Glomerular Filtration Rate (GFR):** We will use the existing CKD-EPI equation to estimate GFR to assess eligibility [11]. Older participants will have lower levels of estimated GFR (eGFR) at enrollment. Many of the AIs speak their Native language along with English. Our community health representatives (CHRs) will help with study related procedures and will administer the consent in patients’ Native language such as Zuni or English depending on participant preferences.

**Exclusion Criteria at Enrollment:** [1] no consent [2]; life expectancy < 3 years [3]; institutionalized subjects [4]; ESKD or renal transplant [5]; renal cancer [6]; myeloma [7]; immunosuppression [8]; autosomal dominant polycystic kidney disease [9]; participation in any other Clinical trial [10]; current pregnancy; or, [11] current incarceration.

### Consent process

All sites participating in this study will use the same basic process to consent participants. The informed consent document will be structured such that it enables potential participants to indicate in which aspects of the study they are willing to engage (e.g., genetic component versus Biologic aspects). The consent process will cover all aspects of screening, baseline testing and subsequent follow-up visits. A separate section and signature page will be required for consent to collect a blood sample for genetic testing and storage of DNA.

Investigators, IRB-approved research coordinators, or CHRs will be responsible for obtaining consent. All personnel obtaining informed consent will have completed Collaborative Institutional Training Initiative (CITI) training, and received training from the principal investigator (PI) for consenting procedures in line with the UHM HSC Human Research Protections Office (HRPO) standard operating procedures (SOP) for Informed Consent titled, “SOP: Written Documentation of Consent (HRP-091)”. We will use a document of informed consent for this study. A copy of the signed/dated informed consent form will be given to every participant.

### Study timeline

Recruitment and patient enrollment will be completed in the first three [3] years of the study. Participant duration in the study may be as long as five years (60 months) if they are recruited in the first year of the study and complete all study visits. Some participants’ study duration will be shorter if they are recruited in Years 2 or 3 of the study.

### Study endpoints

The CRIC Study has a defined series of principal clinical outcome events that will be the focus of the primary longitudinal analyses. These outcomes can be broadly categorized as kidney disease events (ESKD or eGFR decline), cardiovascular events, patient-centered outcomes and death (see Table 1).

**Table 1:** Non-kidney Clinical Outcomes

**Cardiovascular**	● Acute myocardial infarction ● Hospitalization for congestive heart failure ● Serious cardiac arrhythmia
**Peripheral Vascular**	● Amputation for peripheral artery disease ● Surgical or percutaneous revascularization for peripheral artery disease
**Cerebrovascular**	● Intraparenchymal hemorrhage ● Subarachnoid hemorrhage ● Large-vessel cerebral infarction ● Cardioembolic cerebral infarction ● Small-vessel cerebral infarction ● Cerebral infarction not otherwise specified
**Death**	● Atherosclerotic Coronary Heart Disease Death ● Cerebrovascular Death ● Other Atherosclerotic Disease Death ● Other Cardiovascular Disease Death ● Other Death ● Sudden Cardiac Death

### Research setting

#### Research locations

To enhance efficiency and ensure timely recruitment, we established a consortium experienced in conducting studies in AI communities. Participating organizations include the town of Guadalupe and NIDDK office in Phoenix; First Nation hospital and Albuquerque area Indian Health Services (IHS); and Zuni Pueblo, NM.

#### Recruitment methods

We will use the patient panel of CRIC investigators in our recruitment areas (Phoenix, AZ, Zuni, NM and Albuquerque, NM), since our sites have a track record for clinical studies recruitment. We will recruit participants from nephrology, diabetes, and internal medicine clinics. We will contact potential participants in compliance with HIPAA guidelines, guidelines of local IRBs, and the policies of each medical facility. We will approach potential participants either at the time of a visit, when they are referred through health care providers other than AI-CRIC investigators, or when they respond to advertising. We will provide a letter to potential participants that we screened through health care providers’ databases. The invitational letter will be signed by the local principal investigator and/or personal physician. We will also use community outreach, peer-to-peer recruitment, publish informational articles in local newspapers, and participate in local radio health-related broadcasting, to increase our sample size. Finally, the study staff will use mail, phone, or home visits for additional contacts.

We will then schedule those who are interested for a visit or phone call to assess eligibility. After initial screening, we will schedule those who are eligible and remain interested for an in-person screening and informed consent session. We will submit all recruitment materials for prior approval from the Human Research Review Committee (HRRC) of each site before we use them.

#### Community-based research staff

Our research staff will consist of four CHRs with associate degrees in nursing or health/life science. They will have responsibility for recruitment and performing the baseline and follow-up visits.

#### Study procedures

##### Initial pre-screening

AIs frequently live in homes not equipped with landlines, however majority of them carry mobile phones. CHRs will call prospective participants known to have phones based on our previous contacts through our continuous work in these communities. CHRs will also visit the homes and/or workplaces of potential participants. Potential participants may also be approached during scheduled visits to health professionals. If people are un-decided, CHRs will leave contact information and re-contact undecided participants in two weeks. If an eligible individual ultimately refuses participation, the CHR will document this on a refusal form.

##### Screening visit

Currently we have trained CHRs at all sites who will obtain the following: [1] informed consent [2]; demographic and contact information [3]; blood from a finger stick to measure A1C and Glucose using POC instruments [4]; urine to measure protein using a POC instrument; and [5] an eligibility questionnaire.

##### Baseline visit

The baseline study enrollment visit will occur within 45 days of the screening visit. We will follow the parent CRIC protocol [12] for all the methods and processes for the baseline visit including: [1] confirm eligibility [2]; medical history and physical examination [3]; approximately 65 cc of blood for fasting blood sample to measure glucose levels and glucose control, kidney function, cholesterol levels, electrolytes and measures of inflammation, and lymphocytes if consenting to DNA studies [5]; urine sample for protein, albumin, and urea nitrogen [6]; medication record [7]; ankle-brachial index [8]; anthropometrics [9]; questionnaires on dietary intake, physical activity, SF12, depression, cognitive function, and health resource utilization. This visit will last approximately 75 min.

##### Follow-up visits

We expect to follow participants from 30 to 54 months, depending on date of enrolment. We will repeat baseline measurements and observations annually. We will contact all participants six months after each of these visits to update contact information, ascertain interim outcome events, and assess health resource utilization. We will conduct follow-up visits at 11 to 13-month intervals.

##### Monthly finger-sticks

Patients will receive a handheld creatinine monitor (StatSensor Creatinine Monitor, about the size of a cell phone) and all the supplies needed to do finger-stick creatinine testing at home. Patients will be asked to do the testing at home monthly for one year and weekly for two months of that year. At the clinic visit, we will show patients a short video explaining how to do the testing. Afterwards, they will be asked to practice the test in the clinic before they leave to make sure they understand the process and to allow time for them to ask any questions. It will take about 15 min to learn how to perform finger-stick creatinine testing at home. For each test, the patient will first do a “finger-stick” to get a drop of blood from the tip of their finger. They will place the drop of blood on the edge of the testing strip and insert the strip into the meter. The meter will display the creatinine measurement. Once they have measured their creatinine, they will be asked to report the value by calling the AI-CRIC Study. They will discard the test strip after completing the test.

##### Monthly urine albumin testing

patients will receive urine diagnostic dipsticks and scan cards (Scanwell Health Urine Dipsticks and Scan Cards) and will be asked to download the “CRIC at Home” smartphone application. They will be asked to do the testing at home monthly for one year and weekly for two months of that year on the same day they perform the finger-stick creatinine test. At their clinic visit, we will describe the process to them. Patients will be asked to practice the test in the clinic before they leave to make sure they understand the process and to allow time for them to ask any questions. It will take about 15 min to learn how to perform the urine albumin testing at home.

Before performing the urine test, patients will be asked to complete a brief survey on risk factors for sudden changes in kidney function through the “CRIC at Home” smartphone application. For each test, they will first collect a urine sample in a specimen cup. After collecting the urine, patients will dip the urine diagnostic dipstick in the urine and then place on the diagnostic scan card and wait for the colors to develop. Using their smartphone, they will launch the “CRIC at Home” application and it will guide them through the process of taking a picture of the diagnostic scan card. After the application finishes scanning the picture, the test result will be displayed. The image and result are stored on the application. This application is unencrypted; however, only the patient’s result and unique ID are stored on the application and all study information will be removed after completion of the study. Once the patient has tested their urine, they will be asked to report the value by calling the AI-CRIC Study. Patients will discard the dipstick and scan card after completing the test.

We will give a printed schedule to patients at the clinic visit to remind patients when to perform the finger-stick creatinine and urine albumin tests. We may also call patients by telephone or contact them by email to remind them to perform these tests and let us know their results. A short questionnaire will also be given to patients to provide feedback on their experience with these home tests, which can be mailed back to us in a pre-paid envelope.

##### Cardiovascular monitoring

We will ask patients wear up to 2 wireless monitors at two different time points over the course of the study. Both activities are described below.

##### Zephyr BioPatch testing

The Zephyr BioPatch™ is a wireless monitor that can measure changes in heart rate and breathing rate, activity level and position, and Electrocardiogram (EKG) patterns. The BioPatch™ consists of a sensor, known as the BioModule™, which is less than 2 in. in diameter and weighs less than an ounce, and a lightweight plastic patch that holds the BioModuleTM in place. Comfortable and small, the BioPatch™ attaches to traditional disposable EKG electrodes placed on the chest and allows the BioModule™ to be easily snapped in. It is powered by a rechargeable battery. Patients will be asked to wear the BioPatch™ for a total of 48 h during which it will log their activity and heart rate data. At the end of 24 h, patients will need to recharge the sensor for a period of 3 h and then place it back on the chest for an additional 24 h. In addition, during the time they are wearing the device, they will be asked to keep a journal of their sleep and wake times, periods of exercise and any other events that might significantly affect biosensor readings. At the end of this period, they will be asked to return the BioPatch™ and journal. We will provide patients with a pre-paid envelope to return the device at their clinic visit.

##### ZIO® XT patch testing

The ZIO® XT patch is a small, adhesive, water resistant single lead EKG sensor that is placed on the chest to monitor heart rhythm. Patients will be asked to wear the ZIO® XT patch for a total of 14 days during which it will log their EKG patterns. At the end of the 14-day period, they will be asked to remove and return the ZIO® XT patch to iRhythm technologies in the prepaid envelope that will be provided to them at their clinic visit.

### Data and specimen banking

We will store blood and urine samples at a central laboratory at the University of Pennsylvania and at the NIDDK storage facility for future studies of kidney disease, heart disease, and their consequences. They will be connected to study results only by a unique study number, which will be assigned to each participant at the time of consent. The archived blood and urine samples can be used at any time during the period for more studies of kidney and heart disease. It is possible that new tests will be available in the future, which could be useful in understanding kidney and heart disease. Researchers who plan to use archived sample for future scientific study will be required to request and receive all of the necessary approvals or waivers from the NIDDK and CRIC study investigators before using the samples. Samples will only be released to scientists who are qualified and prepared to conduct a research study.

### Data analysis

#### Analytical plan for first aim

##### Overview of methods for statistical analysis

We plan to use standard descriptive statistics to characterize the overall study population and subgroups of interest both at baseline and during follow-up. Summary statistics such as means, medians, standard deviations, and ranges will be produced for measured variables. Frequencies will be tabulated for categorical and ordinal variables. Graphical methods will be used to examine distributions, identify potential influential points, and guide in data transformations. For outcomes collected longitudinally, and to examine associations among various measures, scatterplots and grouped boxplots will be produced to examine assumptions of linearity, symmetry, and homoscedasticity. For outcome-related analyses, we consider two primary types of analyses - failure-time analyses and repeated measures analyses.

##### Sample size and power considerations for first aim

We can calculate power for each of the analysis types for this aim. For brevity, we focus on the analyses that will have the least power to detect differences in outcomes (i.e., other analyses will be able to detect smaller differences). The analyses that will have the lowest power relative to the total sample size are those focusing on failure-time (e.g., CVD and ESRD) effects. For these analyses, power depends on: [1] outcome of interest [2]; baseline progression rate [3]; risk factor distribution [4]; significance level [5]; sample size; and [6] clinically meaningful effect size. Although we expect that there may be a number of factors associated with very large risk differences, we consider two-fold relative differences in risk to be clinically meaningful. Our proposed center will recruit 500 diabetic AI, and our primary analyses will be based on matched comparisons to participants of specific racial/ethnic backgrounds from the entire CRIC cohort. As we compare failure-time outcomes between AIs (*n* = 500) and another racial/ethnic group, we will have 80% power to detect HRs of at least 2.0 as long as we have an average of at least 3 years of follow-up within each group, and the lower-risk group has an annual event rate of at least 0.02 per year. The event rates for CKD progression observed in CRIC are higher than this: we expect greater than 95% power to detect HRs of at least 2.0, and 80% power to detect HRs of at least 1.67. Our power to detect a significant impact of an exposure on the HR comparing the AIs to another group will be lower. Still, if the AI HR is 2.0, with the current non-AI CRIC event rate in the non-AI group, then we will have 80% power to detect a significant impact of an exposure on the HR if the exposure reduces the HR by half – the pre-determined clinically meaningful effect. Therefore, we will have at least 80% power to detect clinically meaningful differences in CKD and CVD progression between AIs and other racial/ethnic groups.

##### Recruitment site (center) effects

There may be systematic differences among participating sites (urban vs reservation base) in subject mix and methods of treatment. However, only some of these differences are likely well-characterized. Therefore, we will need to account for potential center/cluster effects to get appropriate estimates of standard errors, and possibly to control for confounding by center. We will consider several ways to account for center in survival models; by fitting mixed- or fixed-effects models including separate center-specific coefficients, by fitting marginal models, and by using models that stratify on center. For prediction in a wider population, the centers will not themselves be of interest; thus, we will rely primarily on marginal models using the robust variance estimator.

##### Analytical plan for second aim

To characterize potential non-linear patterns of CKD progression, we will implement analyses previously described by Li et al. [13] Briefly, we will initially apply a Bayesian smoothing technique to estimate each patient’s eGFR or proteinuria trajectory as a smooth curve. As such, its slope can be calculated month by month, accommodating a possible change in rate of progression over time [13]. We will then classify patients based on disease trajectory using iterative measures. This can be accomplished using two approaches. First, we plan to use latent class mixed effects models [14, 15] as described above. Second, the Bayesian smoothing technique [13] will allow us to estimate the “most likely” trajectory by the average of the generated Monte Carlo curves and the observed variation in curves around the mean estimated curve.

To quantify intra-individual variability in kidney function or damage, we propose to initially follow the approach previously taken by Al-Aly and colleagues [16]. We will define within-person variability as the coefficient of variation of a regression line fitted to all of the monthly fingerstick creatinine-based eGFR or bimonthly protein measures for each individual participant. The general concept of this approach is to calculate the deviation of the measured creatinine-based eGFR, for example, from its fitted value based on a linear regression model for each participant. This measure of kidney function variability has an advantage because it is normalized to the average value for each individual, which means it is a dimensionless metric that can be readily compared across participants who have different absolute levels of eGFR [16]. Furthermore, to improve the precision of the estimation of the intra-individual variability in kidney function or damage, we can take advantage of using the previously identified non-linear values while calculating variability. Depending on the question of interest, and the focus on either pathophysiology or prediction of disease, we will use logistic, failure-time, repeated measure or predictive analyses as described above. These measures of kidney function or damage will be defined over the course of 1 year for individuals, so the outcome assessment period in these models will begin at the end of the sub-protocol period or AKI episode.

##### Sample size and power considerations for second aim

are based on the event rates in the parent CRIC study. As outlined above, a variety of analyses will be performed. We therefore highlight an analysis of particular interest for this aim: one where we extract classes of specific nonlinear patterns of kidney function from the ambulatory monitoring data and then use those classes to predict differences of eGFR slope in the subsequent year. For these assessments, the clinically meaningful differences that we hope to detect are one-year eGFR changes between two non-linear trajectory groups of 5 ml/min/1.73 m2. Even when comparing two groups of nonlinear kidney function patterns, each comprising 15% of the participants, with 500 participants we will have greater than 90% power to detect differences in one-year changes in eGFR of at least 5.0 ml/min/1.73 m2 between groups. This power approximation was accomplished using a two-sided, 0.05 level, two-sample t-test with groups of 75 individuals each, whose within-person variability of one-year changes in eGFR was no greater than 9.05 ml/min/1/73 m2. This demonstrates that with data from 500 participants we will be able to assess clinically meaningful questions in this specific aim.

##### Analytical plans for third aim

We will examine the distribution of newly-identified features related to heart rate, physical activity and heart rhythm abnormalities and how they change over time. We will fit linear or generalized linear mixed models depending on the type of the measures (either continuous, binary or categorical form). These models naturally handle multiple repeated observations from the same individuals by introducing a within subject correlation structure. We will use these models to compare the distribution of different features across subgroups defined by demographic information and standard and novel CV risk factors. We are also interested in whether these features are risk factors and predictive of future clinical outcomes. We will test the hypothesis whether a candidate predictor is a risk factor for clinical outcomes including CV events and CKD trajectories/progression. We will fit Cox proportional hazards models for survival outcomes, e.g., time to heart failure (HF) and time to halving of eGFR, ESKD, and death. We will adjust for confounding factors, including demographic characteristics and traditional and novel CV risk factors, in a stepwise fashion. More specifically, we will start with a model including the candidate predictors derived from the biosensor data and demographic information only. We will then sequentially add traditional and novel CV risk factors to the model. Statistical issues, including potential violations of the proportional hazards assumption and competing risks due to death, will be handled in the same way as described above. We will also evaluate the added predictability of outcomes beyond standard risk factors. Metrics that will be used to quantify the added predictability include the C statistic, Net Reclassification Index, and Integrated Discrimination Index. Confidence intervals for these metrics will be derived using cross-validation and bootstrap resampling.

Sample size and power considerations for third Aim: are based on the event rates in the parent CRIC study. With 500 participants, we will have 80% power to detect HRs of 1.82, 1.91, or 2.74 for the risk of HF or atrial fibrillation associated with the presence of specific nonlinear patterns of CV health if these patterns are observed in 50, 30% or 10% of participants, respectively. As long as a pattern associated with CV events of CKD progression is present in greater than 10%, we will have statistical power to detect clinically meaningful HRs of 2.0.

## Discussion

Knowledge regarding risk factors and trends in the prevalence of CKD, ESKD and CVD as important causes of premature morbidity and mortality are crucial for health care policy and planning. CKD affects approximately 11 to 13% of adult population worldwide [17]. Novel approaches to identify the role of subclinical risk factors at granular levels for rapid loss of kidney function include the ability to measure the variability of kidney function over time, and to detect occult acute decrements in kidney function [16].

We will test the prevailing paradigm for kidney disease progression that kidney function loss occurs at a relatively stable, linear rate [18, 19]. Several recent reports that have challenged this assumption support our hypothesis. The African American Study of Kidney disease and hypertension (AASK) analyzed up to 30 eGFR readings per participant over 12 years and showed that nonlinear trajectories were quite common. Many participants had substantial periods of stable or increasing eGFR, or substantial periods of rapid decrease in eGFR [20, 21]. Another study demonstrated variable GFR trajectories in Pima Indians [22]and reported no relationship between early GFR and progression of kidney disease. These nonlinear trajectories, which are not commonly assessed in clinical practice may have important prognostic implications. For example, CRIC participants initiating hemodialysis with an antecedent abrupt decline in kidney function (8.5%) experienced, on average, a 3-fold higher risk for death within the first year of dialysis [23]. There is emerging literature suggesting that, independent of long-term GFR trajectory over time, short-term variations in eGFR are predictive of kidney disease outcomes [24–26].

In addition, reduced eGFR and increased albuminuria levels are associated with a higher risk of CVD in patients with mild CKD [27, 28]. CKD and receiving maintenance dialysis are major risk factors for CVD [29], a leading cause of death in the US [30]. IHS data suggest that CVD mortality rates vary greatly among AI communities and appear to be increasing. However, relatively few studies have assessed current disparities in cardiovascular disease in American Indian populations with CKD and compared trends with other regions of the United States [31]. In AI-CRIC, we will seek risk factors for higher rates of CKD and CVD and their progression in Southwest AIs, a socioeconomic disadvantaged population, compared with Caucasians, African Americans and Hispanics participating in CRIC.

Higher mortality rate [32, 33], higher risks of HF [34, 35], heart rate variability (HRV) [36], peripheral arterial disease [37–39], and stroke [40] are reported in patients with CKD. Among CRIC study participants, the overall incidence of HF was 21.7 per 1000 person-years and 18.6% of participants developed incident peripheral arterial disease during 6.3 years of follow-up [41]. Left ventricular hypertrophy is very common in CKD patients. CRIC showed that left ventricular mass index was strongly associated with incident hear failure, even after adjustment for CVD risk factors and biomarkers [42]. Likewise, CRIC reported that low eGFR was strongly associated with greater coronary artery calcification and significantly predicted the risk of CVD [43, 44]. American Indian participants in the SHS had a higher prevalence of sub-clinical cardiac abnormalities on echocardiogram, including left ventricular hypertrophy, left atrial dilation, and reduced left ventricular systolic and diastolic function [45]. HRV in a variety of clinical settings has been used to predict death and HF [46, 47]. To the best of our knowledge, little is known about the prognostic value of biometric monitoring in CKD. In particular, using such monitoring to detect individuals with limited CV reserve prior to a CV event may provide novel opportunities to implement preventive interventions. With AI-CRIC, we will use remote biometric monitoring to measure HRV as an indicator of limited CV reserve. In the CRIC Study even limited data showed that HRV is associated with mortality [48]. Thus, our goal is to test the hypothesis that wearable biosensor technologies can provide robust measures of HRV in CKD patients. This test will enable us to detect a subset of CKD participants at higher risk for HF as candidates to subsequently test the value of intervention programs.

Many previous studies demonstrated the relationship between diabetes, obesity, and hypertension in persons with chronic kidney disease. RAASI treatment correlates with subsequent reduction in the rate of ESKD [49, 50]. The prevalence of hypertension in CKD varies by race, etiology of CKD and GFR [5]. Considering the fact that there are currently no markers available to predict the subsequent loss of kidney function in AI patients already treated with RAASi, in the AI-CRIC study we will investigate such markers.

Among AIs, risk factors such as lower socioeconomic level [51, 52] and exposure to various persistent environmental pollutants [53, 54] become endemic with high rates of obesity, type 2 diabetes, kidney disease, hypertension and CVD. Socioeconomic disadvantage has not yet been identified in clinical guidelines as a high-risk factor warranting screening for CKD, although CKD is associated with socioeconomic deprivation [55]. In the US, ESKD incidence and progression is increasing disproportionately in poorer neighborhoods in comparison with richer ones [52, 56, 57].

Toxic environmental pollutants are frequently found in minority and disadvantaged communities because of their greater proximity to landfills and other industrial waste sites. Environmental exposure to several heavy metals such as tungsten, uranium, arsenic and cadmium are associated with a number of health conditions [54], including diabetes [58], CVD [59], hypertension [60] and irreversible kidney damages [61]. Exposure to various contaminants may also be related to the type of work a person performs. These elements are also found in rural farming populations such as in AIs of the Southwest US. In AIs, agricultural work and jewelry making result in exposure to various heavy metals that may be nephrotoxic. The extent to which environmental/occupational exposures in AIs contribute to progression of CKD and CVD remain poorly understood and further studies are needed. AI_CRIC will investigate whether unique associations between environmental/occupational exposures and the progression of CKD in AIs are partly responsible for these differences. In this study, we will monitor kidney function by conducting monthly home testing of fingerstick creatinine concentration using a handheld device.

Despite the strengths of the study, there are some potential pitfalls and limitations; we recognize that observed monthly changes in eGFR based on fingerstick creatinine measures may not reflect actual changes in underlying kidney function but rather variability in creatinine production. Our plan is to characterize potential non-linear eGFR trajectories over a 12 month testing period. With regards to urine albumin/protein excretion testing using the proposed urine diagnostic testing methods, there are well-known challenges in standardizing and accurately quantifying the amount of albumin or total protein by these methods. Nonetheless, the ability to detect the presence of and changes in semi-quantitative measures of urine albumin or protein has important potential clinical and prognostic implications. Furthermore, despite any measurement limitations, our study will provide key insights into the potential bimonthly variability in semi-quantitative urine albumin/protein excretion within the context of concurrent measures of eGFR. The total period of time during which each participant will wear the monitors is limited by features of the devices used (data storage capacity of the Zephyr BioPatch, and battery life in the ZIO XT Patch). While both devices will be worn long enough to capture diurnal variations, it is possible that some participants will wear the devices during periods in which the type and level of their activities are nonrepresentative. Finally, a larger number of patients may drop out from the study than we considered in the power calculations. The drop-out rate will be monitored and the enrollment target may be adjusted upwards.

In conclusion AI-CRIC study will help us to learn more about ESKD and its progression and relationship with CVD.

### Ethics approval & consent to participate

We received one ethical approval from University of New Mexico Institutional Review Board (UNM-IRB) for all sites participating in this study. We will use the same basic process to consent participants across all sites. The informed consent document will be structured such that it enables potential participants to indicate which aspects of study they may not be willing to engage in (e.g., genetic study). The consent will cover all aspects of screening, baseline testing and subsequent follow-up visits. A separate section and signature page will be required for consent to collect a blood sample for genetic testing and storage of DNA.

Investigators and University of New Mexico Institutional Review Board (UNM-IRB) approved research coordinators and/or Community Health Representatives will be responsible for obtaining consent. All personnel obtaining informed consent will have completed Collaborative Institutional Training Initiative (CITI) training, and received training from the principal investigator (PI) for consenting procedures in line with the UNM-IRB of Human Research Protections Office (HRPO) standard operating procedures (SOP) for Informed Consent titled, “SOP: Written Documentation of Consent (HRP-091)”. A document of informed consent will be used for this study. A copy of the signed/dated informed consent form will be given to the subject.

We obtained Letters of Support from Zuni Comprehensive Community Health Center - Indian Health Services, First Nations Community Health Source, Pueblo of Zuni Tribal Governor, and NIDDK, NIH Phoenix.

## Data Availability

The data that support the findings of this study may be available on request from both the PIs (M. Unruh & V. Shah). We will use reporting standards of STrengthening the Reporting of OBservational studies in Epidemiology (STROB).

## Abbreviations

AKI: Acute Kidney Injury
AI-CRIC: American Indian Chronic Renal Insufficiency Cohort
AI: American Indian
CVD: Cardiovascular disease
CV: Cardiovascular
CKD: Chronic kidney disease
CRIC: Chronic Renal Insufficiency Cohort
CITI: Collaborative Institutional Training Initiative
CHR: Community health representatives
DSMB: Data and Safety Monitoring Board
EKG: Electrocardiogram
ESKD: End stage kidney disease
eGFR: Estimated Glomerular Filtration Rate
ECHO: Extension for Community Healthcare Outcomes
GEEs: Generalized estimating equations
GFR: Glomerular Filtration Rate
HR: Hazard ratios
HIPAA: Health Insurance Portability and Accountability Act
HSC: Health Sciences Center
HRPO: Human Research Protections Office
HRRC: Human Research Review Committee
HF: Heart Failure
HRV: Heart rate variability
IHS: Indian Health Services
IRB: Institutional Review Board
NIDDK: National Institute of Diabetes and Digestive and Kidney Diseases
NIH: National Institutes of Health
PI: Principal investigator
RAASi: Renin-angiotensin-aldosterone system inhibitors
RPMS: Resource and Patient Management System
SOP: Standard operating procedures
US: United States
UNM: University of New Mexico
UNMHSC: University of New Mexico Health Science Center
UACR: Urine Albumin-to-Creatinine Ratio
USRDS: US Renal Data System

## References

[1] Coresh J (2017). Update on the burden of CKD. J Am Soc Nephrol.

[2] Saran R, Robinson B, Abbott KC, Agodoa LYC, Bragg-Gresham J, Balkrishnan R, Bhave N, Dietrich X, Ding Z, Eggers PW, Gaipov A, Gillen D, Gipson D, Gu H, Guro P, Haggerty D, Han Y, He K, Herman W, Heung M, Hirth RA, Hsiung JT, Hutton D, Inoue A, Jacobsen SJ, Jin Y, Kalantar-Zadeh K, Kapke A, Kleine CE, Kovesdy CP, Krueter W, Kurtz V, Li Y, Liu S, Marroquin MV, McCullough K, Molnar MZ, Modi Z, Montez-Rath M, Moradi H, Morgenstern H, Mukhopadhyay P, Nallamothu B, Nguyen DV, Norris KC, O'Hare AM, Obi Y, Park C, Pearson J, Pisoni R, Potukuchi PK, Repeck K, Rhee CM, Schaubel DE, Schrager J, Selewski DT, Shamraj R, Shaw SF, Shi JM, Shieu M, Sim JJ, Soohoo M, Steffick D, Streja E, Sumida K, Kurella Tamura M, Tilea A, Turf M, Wang D, Weng W, Woodside KJ, Wyncott A, Xiang J, Xin X, Yin M, You AS, Zhang X, Zhou H, Shahinian V, Renal Data System US (2019). 2018 Annual Data report: epidemiology of kidney disease in the United States. Am J Kidney Dis.

[3] Yracheta JM, Lanaspa MA, Le MT, Abdelmalak MF, Alfonso J, Sanchez-Lozada LG, Johnson RJ (2015). Diabetes and kidney disease in American Indians: potential role of sugar-sweetened beverages. Mayo Clin Proc.

[4] Tanamas SK, Reddy SP, Chambers MA, Clark EJ, Dunnigan DL, Hanson RL, Nelson RG, Knowler WC, Sinha M (2018). Effect of severe obesity in childhood and adolescence on risk of type 2 diabetes in youth and early adulthood in an American Indian population. Pediatr Diabetes.

[5] Shara NM, Wang H, Mete M, Al-Balha YR, Azalddin N, Lee ET, Franceschini N, Jolly SE, Howard BV, Umans JG (2012). Estimated GFR and incident cardiovascular disease events in American Indians: the strong heart study. Am J Kidney Dis.

[6] Bullock A, Burrows NR, Narva AS, Sheff K, Hora I, Lekiachvili A, Cain H, Espey D (2017). Vital signs: decrease in incidence of diabetes-related end-stage Renal disease among American Indians/Alaska natives - United States, 1996-2013. MMWR Morb Mortal Wkly Rep.

[7] Heerspink HJ, Kropelin TF, Hoekman J, de Zeeuw D (2015). Drug-induced reduction in albuminuria is associated with subsequent Renoprotection: a meta-analysis. J Am Soc Nephrol.

[8] Wright JT, Williamson JD, Whelton PK, Snyder JK, Sink KM, Rocco MV, Reboussin DM, Rahman M, Oparil S, Lewis CE, Kimmel PL, Johnson KC, Goff DC, Fine LJ, Cutler JA, Cushman WC, Cheung AK, Ambrosius WT (2015). A randomized trial of intensive versus standard blood-pressure control. N Engl J Med.

[9] Cooper CJ, Murphy TP, Cutlip DE, Jamerson K, Henrich W, Reid DM, Cohen DJ, Matsumoto AH, Steffes M, Jaff MR, Prince MR, Lewis EF, Tuttle KR, Shapiro JI, Rundback JH, Massaro JM, D'Agostino RB, Dworkin LD (2014). Stenting and medical therapy for atherosclerotic renal-artery stenosis. N Engl J Med.

[10] Rahman M, Pressel S, Davis BR, Nwachuku C, Wright JT, Whelton PK, Barzilay J, Batuman V, Eckfeldt JH, Farber MA, Franklin S, Henriquez M, Kopyt N, Louis GT, Saklayen M, Stanford C, Walworth C, Ward H, Wiegmann T (2006). Cardiovascular outcomes in high-risk hypertensive patients stratified by baseline glomerular filtration rate. Ann Intern Med.

[11] Levey AS, Stevens LA, Schmid CH, Zhang YL, Castro AF, Feldman HI, Kusek JW, Eggers P, Van Lente F, Greene T, Coresh J (2009). A new equation to estimate glomerular filtration rate. Ann Intern Med.

[12] Feldman HI, Appel LJ, Chertow GM, Cifelli D, Cizman B, Daugirdas J, Fink JC, Franklin-Becker ED, Go AS, Hamm LL, He J, Hostetter T, Hsu CY, Jamerson K, Joffe M, Kusek JW, Landis JR, Lash JP, Miller ER, Mohler ER, Muntner P, Ojo AO, Rahman M, Townsend RR, Wright JT (2003). The chronic Renal insufficiency cohort (CRIC) study: design and methods. J Am Soc Nephrol.

[13] Beck GJ, Berg RL, Coggins CH, Gassman JJ, Hunsicker LG, Schluchter MD, Williams GW (1991). Design and statistical issues of the modification of diet in Renal disease trial. The modification of diet in Renal disease study group. Control Clin Trials.

[14] Verbeke G, Lesaffre E (1996). A linear mixed-effects model with heterogeneity in the random-effects population. J Am Stat Assoc.

[15] Proust-Lima C, Philipps V, Liquet B (2015). Estimation of extended mixed models using latent classes and latent processes: the R package lcmm.

[16] Al-Aly Z, Balasubramanian S, McDonald JR, Scherrer JF, O'Hare AM (2012). Greater variability in kidney function is associated with an increased risk of death. Kidney Int.

[17] Hill NR, Fatoba ST, Oke JL, Hirst JA, O'Callaghan CA, Lasserson DS, Hobbs FD (2016). Global prevalence of chronic kidney disease - a systematic review and meta-analysis. PLoS One.

[18] Lewis J, Gonzales M, Burnette C, Benally M, Seanez P, Shuey C, Nez H, Nez C, Nez S (2015). Environmental exposures to metals in native communities and implications for child development: basis for the Navajo birth cohort study. J Soc Work Disabil Rehabil.

[19] Gonzales M, Shah V, Bobelu A, Qualls C, Natachu K, Bobelu J, Jamon E, Neha D, Paine S, Zager P (2004). Concentrations of surface-dust metals in native American jewelry-making homes in Zuni Pueblo, New Mexico. Arch Environ Health.

[20] Weldegiorgis M, de Zeeuw D, Li L, Parving HH, Hou FF, Remuzzi G, Greene T, Heerspink HJL (2018). Longitudinal estimated GFR trajectories in patients with and without type 2 diabetes and nephropathy. Am J Kidney Dis.

[21] Li L, Astor BC, Lewis J, Hu B, Appel LJ, Lipkowitz MS, Toto RD, Wang X, Wright JT, Greene TH (2012). Longitudinal progression trajectory of GFR among patients with CKD. Am J Kidney Dis.

[22] Lemley KV, Boothroyd DB, Blouch KL, Nelson RG, Jones LI, Olshen RA, Myers BD (2005). Modeling GFR trajectories in diabetic nephropathy. Am J Physiol Renal Physiol.

[23] Hsu RK, Chai B, Roy JA, Anderson AH, Bansal N, Feldman HI, Go AS, He J, Horwitz EJ, Kusek JW, Lash JP, Ojo A, Sondheimer JH, Townsend RR, Zhan M, Hsu CY (2016). Abrupt decline in kidney function before initiating hemodialysis and all-cause mortality: the chronic Renal insufficiency cohort (CRIC) study. Am J Kidney Dis.

[24] Tseng CL, Lafrance JP, Lu SE, Soroka O, Miller DR, Maney M, Pogach LM (2015). Variability in estimated glomerular filtration rate values is a risk factor in chronic kidney disease progression among patients with diabetes. BMC Nephrol.

[25] Uehara K, Yasuda T, Shibagaki Y, Kimura K (2015). Estimated glomerular filtration rate variability independently predicts Renal prognosis in advanced chronic kidney disease patients. Nephron.

[26] Choi HY, Huh KH, Lee JG, Song MK, Kim MS, Kim YS, Kim BS (2016). Variability of the estimated glomerular filtration rate in the first year after kidney transplantation is an independent risk factor for poor Renal allograft outcomes: a retrospective cohort study. PLoS One.

[27] Tanaka F, Komi R, Makita S, Onoda T, Tanno K, Ohsawa M, Itai K, Sakata K, Omama S, Yoshida Y, Ogasawara K, Ishibashi Y, Kuribayashi T, Okayama A, Nakamura M (2016). Low-grade albuminuria and incidence of cardiovascular disease and all-cause mortality in nondiabetic and normotensive individuals. J Hypertens.

[28] Matsushita K, van der Velde M, Astor BC, Woodward M, Levey AS, de Jong PE, Coresh J, Gansevoort RT (2010). Association of estimated glomerular filtration rate and albuminuria with all-cause and cardiovascular mortality in general population cohorts: a collaborative meta-analysis. Lancet.

[29] Poli FE, Gulsin GS, McCann GP, Burton JO, Graham-Brown MP (2019). The assessment of coronary artery disease in patients with end-stage renal disease. Clin Kidney J.

[30] Correction to: Heart Disease and Stroke Statistics-2017 Update (2017). A Report From the American Heart Association. Circulation.

[31] Harwell TS, Oser CS, Okon NJ, Fogle CC, Helgerson SD, Gohdes D (2005). Defining disparities in cardiovascular disease for American Indians: trends in heart disease and stroke mortality among American Indians and whites in Montana, 1991 to 2000. Circulation.

[32] Eastwood SV, Chaturvedi N, Sattar N, Welsh PI, Hughes AD, Tillin T. Impact of kidney function on cardiovascular risk and mortality: a comparison of south Asian and European cohorts. Am J Nephrol. 2019;50:425–433. 10.1159/000503873 PMC6892637 31665726

[33] Amod A, Buse JB, McGuire DK, Pieber TR, Pop-Busui R, Pratley RE, Zinman B, Hansen MB, Jia T, Mark T, Poulter NR. Glomerular filtration rate and associated risks of cardiovascular events, mortality, and severe hypoglycemia in patients with type 2 diabetes: secondary analysis (DEVOTE 11). Diabetes Ther. 2020;11:53–70. 10.1007/s13300-019-00715-x PMC6974100 31667706

[34] Mavrakanas TA, Khattak A, Wang W, Singh K, Charytan DM (2019). Association of Chronic Kidney Disease with preserved ejection fraction heart failure is independent of baseline cardiac function. Kidney Blood Press Res.

[35] Alcober-Morte L, Barrio-Ruiz C, Parellada-Esquius N, Subirana I, Comin-Colet J, Grau M, Degano IR, Cainzos-Achirica M, Cunillera-Puertolas O, Cobo-Guerrero S, Mestre-Ferrer J, Pascual-Benito L, Cerain-Herrero MJ, Gil-Terron N, Rodriguez-Latre L, Tamayo-Ojeda C, Salvador-Gonzalez B. Heart failure admission across glomerular filtration rate categories in a community cohort of 125,053 individuals over 60 years of age. Hypertens Res. 2019;42(12):2013–2020. 10.1038/s41440-019-0315-6 31477871

[36] Chou YH, Huang WL, Chang CH, Yang CCH, Kuo TBJ, Lin SL, Chiang WC, Chu TS (2019). Heart rate variability as a predictor of rapid renal function deterioration in chronic kidney disease patients. Nephrology (Carlton).

[37] Subramanian N, Xu J, Sayyed Kassem L, Simonson M, Desai N (2019). Absent or diminished pedal pulses and estimated GFR decline in patients with diabetic kidney disease. Ren Fail.

[38] Kim ED, Tanaka H, Ballew SH, Sang Y, Heiss G, Coresh J, Matsushita K (2018). Associations between kidney disease measures and regional pulse wave velocity in a large community-based cohort: the atherosclerosis risk in communities (ARIC) study. Am J Kidney Dis.

[39] Matsushita K, Ballew SH, Coresh J, Arima H, Arnlov J, Cirillo M, Ebert N, Hiramoto JS, Kimm H, Shlipak MG, Visseren FLJ, Gansevoort RT, Kovesdy CP, Shalev V, Woodward M, Kronenberg F (2017). Measures of chronic kidney disease and risk of incident peripheral artery disease: a collaborative meta-analysis of individual participant data. Lancet Diabetes Endocrinol.

[40] Toyoda K, Ninomiya T (2014). Stroke and cerebrovascular diseases in patients with chronic kidney disease. Lancet Neurol.

[41] Chen J, Mohler ER, Xie D, Shlipak M, Townsend RR, Appel LJ, Ojo A, Schreiber M, Nessel L, Zhang X, Raj D, Strauss L, Lora CM, Rahman M, Hamm LL, He J (2016). Traditional and non-traditional risk factors for incident peripheral arterial disease among patients with chronic kidney disease. Nephrol Dial Transplant.

[42] Dubin RF, Deo R, Bansal N, Anderson AH, Yang P, Go AS, Keane M, Townsend R, Porter A, Budoff M, Malik S, He J, Rahman M, Wright J, Cappola T, Kallem R, Roy J, Sha D, Shlipak MG (2017). Associations of conventional echocardiographic measures with incident heart failure and mortality: the chronic Renal insufficiency cohort. Clin J Am Soc Nephrol.

[43] Bundy JD, Cai X, Scialla JJ, Dobre MA, Chen J, Hsu CY, Leonard MB, Go AS, Rao PS, Lash JP, Townsend RR, Feldman HI, de Boer IH, Block GA, Wolf M, Smith ER, Pasch A, Isakova T (2019). Serum calcification propensity and coronary artery calcification among patients with CKD: the CRIC (chronic Renal insufficiency cohort) study. Am J Kidney Dis.

[44] Budoff MJ, Rader DJ, Reilly MP, Mohler ER, Lash J, Yang W, Rosen L, Glenn M, Teal V, Feldman HI (2011). Relationship of estimated GFR and coronary artery calcification in the CRIC (chronic Renal insufficiency cohort) study. Am J Kidney Dis.

[45] Chinali M, de Simone G, Roman MJ, Best LG, Lee ET, Russell M, Howard BV, Devereux RB (2008). Cardiac markers of pre-clinical disease in adolescents with the metabolic syndrome: the strong heart study. J Am Coll Cardiol.

[46] Al-Zaiti SS, Pietrasik G, Carey MG, Alhamaydeh M, Canty JM, Fallavollita JA (2019). The role of heart rate variability, heart rate turbulence, and deceleration capacity in predicting cause-specific mortality in chronic heart failure. J Electrocardiol.

[47] Fang SC, Wu YL, Tsai PS. Heart rate variability and risk of all-cause death and cardiovascular events in patients with cardiovascular disease: a meta-analysis of cohort studies. Biol Res Nurs. 2019. 10.1177/1099800419877442. 10.1177/1099800419877442 31558032

[48] Drawz PE, Babineau DC, Brecklin C, He J, Kallem RR, Soliman EZ, Xie D, Appleby D, Anderson AH, Rahman M (2013). Heart rate variability is a predictor of mortality in chronic kidney disease: a report from the CRIC study. Am J Nephrol.

[49] Tanamas SK, Hanson RL, Nelson RG, Knowler WC (2017). Effect of different methods of accounting for antihypertensive treatment when assessing the relationship between diabetes or obesity and systolic blood pressure. J Diabetes Complicat.

[50] Nistor I, De Sutter J, Drechsler C, Goldsmith D, Soler MJ, Tomson C, Wiecek A, Donciu MD, Bolignano D, Van Biesen W, Covic A (2018). Effect of renin-angiotensin-aldosterone system blockade in adults with diabetes mellitus and advanced chronic kidney disease not on dialysis: a systematic review and meta-analysis. Nephrol Dial Transplant.

[51] Crews DC, Gutierrez OM, Fedewa SA, Luthi JC, Shoham D, Judd SE, Powe NR, McClellan WM (2014). Low income, community poverty and risk of end stage renal disease. BMC Nephrol.

[52] Garrity BH, Kramer H, Vellanki K, Leehey D, Brown J, Shoham DA (2016). Time trends in the association of ESRD incidence with area-level poverty in the US population. Hemodial Int.

[53] Pang Y, Peng RD, Jones MR, Francesconi KA, Goessler W, Howard BV, Umans JG, Best LG, Guallar E, Post WS, Kaufman JD, Vaidya D, Navas-Acien A (2016). Metal mixtures in urban and rural populations in the US: the multi-ethnic study of atherosclerosis and the strong heart study. Environ Res.

[54] Grau-Perez M, Zhao J, Pierce B, Francesconi KA, Goessler W, Zhu Y, An Q, Umans J, Best L, Cole SA, Navas-Acien A, Tellez-Plaza M (2019). Urinary metals and leukocyte telomere length in American Indian communities: the strong heart and the strong heart family study. Environ Pollut.

[55] Vart P, Gansevoort RT, Joosten MM, Bultmann U, Reijneveld SA (2015). Socioeconomic disparities in chronic kidney disease: a systematic review and meta-analysis. Am J Prev Med.

[56] Nicholas SB, Kalantar-Zadeh K, Norris KC (2015). Socioeconomic disparities in chronic kidney disease. Adv Chronic Kidney Dis.

[57] Nee R, Yuan CM, Hurst FP, Jindal RM, Agodoa LY, Abbott KC (2017). Impact of poverty and race on pre-end-stage renal disease care among dialysis patients in the United States. Clin Kidney J.

[58] Kuo CC, Moon K, Thayer KA, Navas-Acien A (2013). Environmental chemicals and type 2 diabetes: an updated systematic review of the epidemiologic evidence. Curr Diab Rep.

[59] Tellez-Plaza M, Guallar E, Navas-Acien A (2018). Environmental metals and cardiovascular disease. Bmj.

[60] Franceschini N, Fry RC, Balakrishnan P, Navas-Acien A, Oliver-Williams C, Howard AG, Cole SA, Haack K, Lange EM, Howard BV, Best LG, Francesconi KA, Goessler W, Umans JG, Tellez-Plaza M (2017). Cadmium body burden and increased blood pressure in middle-aged American Indians: the strong heart study. J Hum Hypertens.

[61] Charania NA, Tsuji LJ, Martin ID, Liberda EN, Cote S, Ayotte P, Dewailly E, Nieboer E (2014). An examination of traditional foods and cigarette smoking as cadmium sources among the nine first nations of Eeyou Istchee, Northern Quebec, Canada. Environ Sci Process Impacts.

